# Rice Byproduct Compounds: From Green Extraction to Antioxidant Properties

**DOI:** 10.3390/antiox13010035

**Published:** 2023-12-23

**Authors:** Raffaella Colombo, Giulia Moretto, Marta Barberis, Ilaria Frosi, Adele Papetti

**Affiliations:** 1 Department of Drug Sciences, University of Pavia, Viale Taramelli 12, 27100 Pavia, Italy; raffaella.colombo@unipv.it (R.C.); giulia.moretto01@universitadipavia.it (G.M.); marta.barberis01@universitadipavia.it (M.B.); ilaria.frosi01@universitadipavia.it (I.F.); 2 Center for Colloid and Surface Science (C.S.G.I.), Viale Taramelli 12, 27100 Pavia, Italy

**Keywords:** rice byproducts, bioactives, antioxidant compounds, phenolic acids, flavonoids, vitamin E, green methodologies, innovative extraction techniques, structure-activity relationship, functional ingredients

## Abstract

Currently, rice (*Oryza sativa* L.) production and consumption is increasing worldwide, and many efforts to decrease the substantial impact of its byproducts are needed. In recent years, the interest in utilizing rice kernels, husk, bran, and germ for the recovery of different molecules, from catalysts (to produce biodiesel) to bioactive compounds, has grown. In fact, rice byproducts are rich in secondary metabolites (phenolic compounds, flavonoids, and tocopherols) with different types of bioactivity, mainly antioxidant, antimicrobial, antidiabetic, and anti-inflammatory, which make them useful as functional ingredients. In this review, we focus our attention on the recovery of antioxidant compounds from rice byproducts by using innovative green techniques that can overcome the limitations of traditional extraction processes, such as their environmental and economic impact. In addition, traditional assays and more innovative methodologies to evaluate the antioxidant activity are discussed. Finally, the possible molecular mechanisms of action of the rice byproduct antioxidant compounds (phenolic acids, flavonoids, γ-oryzanol, and vitamin E) are discussed as well. In the future, it is expected that rice byproduct antioxidants will be important food ingredients that reduce the risk of the development of several human disorders involving oxidative stress, such as metabolic diseases, inflammatory disorders, and cancer.

## 1. Introduction

Today, recycling and reuse policies represent the key challenge in a global plan for sustainable development. Over the years, this issue has been raised alongside the increase in food processing and consequent increases in food losses and waste worldwide. In fact, 1.3 billion tons, one third of global food production, are lost or wasted every year, making the amount of food waste a global emergency [1]. In addition, the problem of food waste should be evaluated throughout the entire food supply chain, with integrated policies related to all stages, from agriculture to food processing, to prevent environmental damage, from pollution to climate change [1,2,3].

The recovery of food waste and byproducts and their transformation into valuable compounds and healthy products may be the key issue in the circular economy, with various applications in food industries. In particular, cereal, fruit, and vegetable byproducts are rich in high-value compounds, such as phenolics, carotenoids, phytosterols, polysaccharides, proteins, and fatty acids, and their bioactivities can have important beneficial properties in the management of chronic degenerative diseases [3]. The search for innovative technologies to be used in the development of food products derived from plant-based food byproducts is currently an important topic. It is necessary to transform byproducts into valuable substances, as their impact on the current food economy is substantial; therefore, it is important to reuse these wastes by adopting procedures with moderate costs in comparison to the costs of their disposal [1].

Rice (*Oryza* spp.) is one of the main cereals produced worldwide, together with maize and wheat [4]. Over the last several years, rice consumption has seen a slight global increase, especially in East and South Asia and in Latin America, amounting to about 500–600 million metric annual tons. Rice makes a significant caloric contribution to the human diet and contains a wide variety of components with health-promoting properties. Its nutritional content includes carbohydrates (80%), proteins (7–8%), fats (3%), minerals (6–7%), and fiber (3%); in addition, it is rich in bioactive compounds, such as polyphenols, tocopherols, tocotrienols, etc. The main beneficial effect of rice has been long considered its content of antioxidant components, which have a positive effect on the prevention and treatment of diseases in which oxidative stress and inflammation are involved (mainly cardiovascular diseases, metabolic disorders, and neurodegenerative diseases) [5].

The kernel represents 70% of the rice grain, while rice byproducts consist of husk, named also hull (20%), bran (8%), and germ (2%). As rice consumption increases, byproducts recovery is becoming an increasingly important global issue [6,7,8]. Many components extracted from rice byproducts may be used in the food, cosmetic, and pharmaceutical fields, as well as in biofuel processing and production. In fact, cereal byproducts represent an important low-cost source of compounds and are an example of high waste valorization [7,8,9,10,11,12]. The health-promoting properties of rice byproducts arise from the presence of many bioactive molecules, such as vitamins, minerals, fiber, and phenolic compounds with potential applications as ingredients for fortified food and/or food supplements, which would mainly exploit their antioxidant activity (AOA). In fact, the addition of plant-based byproducts represents a suitable strategy by which to develop new ingredients, thus increasing the nutritional value of food products while also improving consumers’ organoleptic perceptions of the food and simultaneously reducing food waste and ecological/environmental pollution [7,8,10]. 

**Figure 1:**
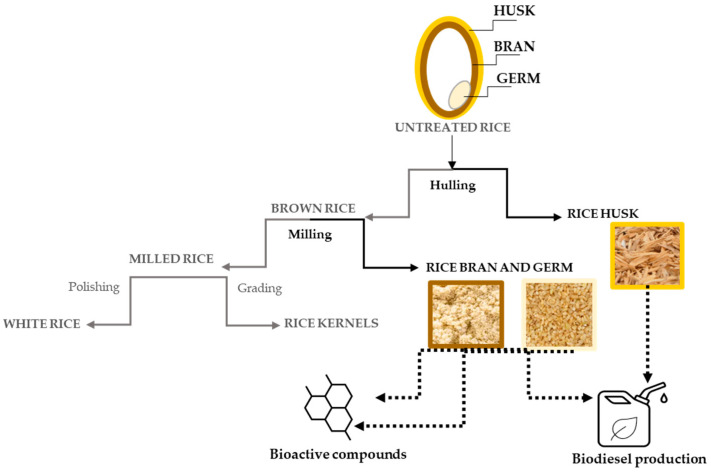
A schematic representation of the processing of rice byproducts and their main applications. The parts in black concern the byproducts discussed herein.

In this review, we discuss the potential applications of rice byproducts (Figure 1), with a particular focus on bioactive compounds showing antioxidant properties, i.e., polyphenols, phenolic acids, and tocopherols. Polyphenols are well known for their potential as common ingredients in functional foods to prevent many disorders, as their AOA has been associated with reductions in cardiovasculopathic, neurodegenerative, inflammatory, and carcinogenic processes [11]. The first aim of this manuscript was to summarize advanced “green extraction” methodologies, as they represent a solution completely in accordance with sustainable approaches in the frame of a global plan of environmental protection. In addition, the traditional analytical methods and more advanced potential methods used to identify and measure AOA were reported. Structure-activity relationship (SAR) studies were also discussed to explore the specific structural characteristics of the antioxidant molecules in association with their bioactivity. This review mainly covers the last decade of literature on the topic.

## 2. Rice Byproducts

### 2.1. Chemical Composition

Rice husk represents the main agro-waste from rice production. It is composed of hemicellulose (28.6%), celluloses (28%), lignin (24.4%), and extractive matter (18.4%) [12,13]. It mainly contains polyphenols possessing well-known antioxidant properties and fibers that have an effect on blood glucose and lipid levels. A potential antiproliferative effect, which is still to be completely explored, on ovarian and colon cancer is probably due to its peptide content [7,14,15]. In addition, rice husk also contains lipophilic compounds (~2%), mainly fatty acids, acylglycerols, and sterols, but their potential remains unexplored [16]. Despite its noteworthy content of bioactive compounds, it is mainly used as feedstock and as a source of catalysts for biodiesel production because of its size, low digestibility, and abrasive properties, which are mainly due to its high silica content (20%). Therefore, although rice husk is important because it protects the seed from oxidation, it mainly remains inedible and is used only in non-food applications [17]. Its high abundance and low cost are two important advantages, which together with its reuse with high yields in biodiesel processing and production, make it a cheap and safe resource that may contribute substantially to the circular economy, considering, for example, that India alone produces over 55 million tons each year of this byproduct [18]. Advanced procedures can be applied to fully use rice husk, first extracting phytochemicals, then producing cellulose nanocrystals that are useful as coating materials from residues [19,20,21].

Rice bran (the outer layer of the rice grain) is derived from the dry-milling process used to obtain white rice, and its global production amounts to 29.3 million tons/year. It is rich in bioactive compounds, such as sterols, essential fatty acids, fibers, tocopherols (mainly γ-tocopherol), tocotrienols, and peptides. It is commonly used in animal feed or to extract rice bran oil (RBO), but the bioactives it contains, especially γ-oryzanol, has been reported to have important antioxidant, anti-inflammatory, hypocholesterolemic, antidiabetic, and anticancer properties [22,23,24,25]. 

Antioxidant, antihypertensive, antidiabetic, and anticancer activities have also been reported for rice-bran peptides, although the research is still at the beginning stages [26].

Colored rice bran contains about one hundred antioxidant molecules. In fact, in addition to γ-oryzanol and tocopherols, it contains ferulic, gallic, protocatechuic, hydroxybenzoic, *p*-coumaric, and sinapic acids, as well as cyanidins [5,27].

Although rice bran is rich in bioactive compounds, its reuse to extract these compounds is usually limited by its instability in the presence of enzymes such as lipase and lipoxidase; therefore, stabilization by germination or parboiling procedures is generally required to maintain high amount levels of oryzanol and tocotrienol [28].

Today, rice bran is widely added to products such as bread, thus not only increasing the products’ content of vitamins B, fibers, and minerals, but also modifying bread textural properties, enhancing hardness, gumminess, and chewiness [29]. 

Rice bran is also a good source of extractable oil, and this oil has been widely studied. The definition of RBO as a byproduct remains controversial; while most RBO is used as a byproduct for non-edible or edible applications, RBO itself represents a nutritious edible product [30]. RBO has a lower phytosterol and tocochromanol content than commercial oils, but it contains a high amount of tocotrienols [31]. Human consumption of RBO is not widespread because it contains high levels of free fatty acids (mainly triacylglycerols); conversely, it has been exploited as an economical source of biodiesel. In fact, the selection of appropriate catalysts applied under the correct concentrations, along with the choice of a suitable alcohol-to-oil ratio, temperature, and duration, makes it possible to obtain yields higher than 80% in a short time (from 20 min to 3 h) [18].

Rice germ is five times richer in vitamin E (mainly α-tocopherol) than rice bran, exhibiting a high antioxidant effect, but it is poorer in γ-oryzanol. It also contains B-group vitamins (B1, B2 and B6), minerals (cadmium, iron, and magnesium), fibers, and γ-aminobutyric acid (GABA), and has an important anticarcinogenic effect [7,32]. This byproduct remains little-explored, but its considerable nutritional value means that it deserves study. Studies should focus on the recovery of peptides, considering its high protein content (18 g/100 g), which is rich in essential amino acids (mainly lysine, histidine, and valine). In addition, its content of fatty acids (linoleic and linolenic acids) is very promising [33].

### 2.2. Focus on the Antioxidant Compounds Present in Rice Byproducts 

As mentioned above, rice byproducts are a rich source of antioxidant compounds, such as phenolic acids, flavonoids, vitamin E, and γ-oryzanol [34]. These compounds have attracted interest due to their ability to counteract oxidative stress, which negatively affects several cellular structures, such as membranes, lipids, proteins, lipoproteins, and deoxyribonucleic acid (DNA) [35]. Phytochemical analysis of rice bran revealed high levels of antioxidant molecules, mainly tocopherol, tocotrienol, γ-oryzanol, and of phenolic compounds distributed in free, soluble-conjugated, and bound forms [5]. Phenolic acids, flavonoids, and anthocyanins, especially in pigmented rice, are the main polyphenolic compounds [27], and ferulic and *p*-coumaric acids have been identified as the most abundant hydroxycinnamic acids in the bran of most rice varieties (black, red, brown, and white) [36], followed by protocatechuic, syringic, and cinnamic acids [37]. Among flavonoids, apigenin, luteolin, quercetin, myricetin, and catechin have been detected. In particular, black rice bran has the highest content of free and bound *p*-coumaric acid, as well as of the free and bound forms of apigenin and quercetin [38]. In addition, among the other phenolic acids, vanillic, sinapic acid, and 4-hydroxybenzoic acids have been identified in red, white [39], black [40], and purple rice bran [41]. In more distinctive findings, isoferulic and gallic acids have been detected in a red variety [39], while black and purple varieties are rich in caffeic and chlorogenic acids [41]. Similarly, Ti et al. identified seven phenolic compounds (gallic, protocatechuic, ferulic, *p*-coumaric, syringic, caffeic, and chlorogenic acids) in the bran of different *indica* rice varieties, confirming that *p*-coumaric and ferulic acids are the most abundant [42]. Interestingly, protocatechualdehyde has been identified in red rice bran. This compound is a naturally occurring compound resulting from phenolic-acid degradation and is well known for its antioxidant properties [39,43]. Considering other classes of antioxidant polyphenols, several studies performed by Peanparkdee et al. confirmed the presence of rutin, a flavonoid compound, in colored and non-colored rice bran extracts obtained from different Thai rice cultivars [34,44]. Another flavonoid compound that has recently generated interest because its beneficial properties is tricin. Different amounts of tricin have been observed in bran from rice with different pericarp colors, with the purple pericarp genotype showing the highest level, mainly due to genetic factors, rather than environmental conditions [45]. Pigmented rice has a high content of anthocyanins, which are responsible for red, brown, purple, and black colors and for many biological effects due to their antioxidant properties [46]. To date, about eighteen different anthocyanins have been detected, although cyanidin 3-*O*-glucoside, peonidin-3-*O*-glucoside, cyanidin-3-*O*-rutinoside, and cyanidin-3-*O*-galactoside are the most frequently identified [47]. As reported by Pang et al. cyanidin-3-*O*-glucoside and peonidin-3-*O*-glucoside are the main anthocyanins present in black rice. These compounds accumulate in the outer layer, making the black rice bran a richer source than whole black grains and giving the bran a greater antioxidant capacity [27]. In fact, bran anthocyanins account for 97% of the total anthocyanins in black rice grains [48]. 

In addition, the nonsaponifiable lipidic fraction of rice bran is rich in functional components, such as γ-oryzanol, tocopherols, and tocotrienols. γ-oryzanol is a mixture of antioxidant compounds, including steryl ferulate and triterpene alcohols. It is mainly composed (95%) of 24-methylenecycloartanyl ferulate, cycloartanyl ferulate, campesteryl ferulate, and β-sitosteryl ferulate [49]. On the other hand, a metabolite profile revealed the presence of different tocopherols and tocotrienols, such as α-tocotrienol, γ-tocotrienol, α-tocopherol, δ-tocopherol, γ-tocopherol, β-tocopherol, which are the main antioxidants present in rice bran [50]. In fact, rice bran is the part of the rice grain richest in vitamin E, particularly tocotrienols (especially γ-tocotrienol) [36]. These compounds constitute the lipid fraction of rice bran, from which RBO is obtained. RBO is also composed of a saponifiable fraction that is made up of triglycerides, diglycerides, monoglycerides, glycolipids, phospholipids, unsaturated fatty acids (such as oleic and linoleic acids), and saturated fatty acids (such as palmitic and stearic acids) [25,51]. Therefore, RBO has been recognized by the World Health Organization (WHO) as a “healthy oil” due to its well-balanced fatty-acids content and its high concentration of compounds responsible for AOA [52]. Thus, all compounds present in rice bran, not only its polyphenols, contribute to AOA. This conclusion is demonstrated by the fact that it has more activity than rice husk, which has a similar phenolic composition but lacks tocopherols, tocotrienols, and γ-oryzanol. 

In rice husk, phenolic acids are mainly present in the bound form, and *p*-coumaric acid, ferulic acid, and vanillic acid (in descending order of abundance), are the main bound phenolic compounds identified in it, as well as in glutinous and roasted rice husk [53,54,55]. Other compounds, such as gallic acid, *p*-hydroxybenzaldehyde, *p*-hydroxybenzoic acid, syringic acid, and cinnamic acid, have been detected in rice husk from several rice varieties [56]. Among flavonoids, apigenin with a pentosyl and glycosyl moiety and tricin have been identified [15]. 

Finally, the chemical characterization of several rice varieties, such as *Indica* and *Japonica*, showed that rice bran and rice husk contain 90% of the total phenolic compounds, providing strong antioxidant activity. A strong correlation between rice varieties, phytochemical composition and antioxidant properties is evident, as shown by the higher phenolic content and antioxidant activity of *Japonica* rice compared with *Indica* rice [56]. 

## 3. Green Methodologies for Rice Byproducts Processing

### 3.1. Pretreatments

Pretreatments, mainly thermal processes (freeze/oven-drying) or chemical procedures (solid-state fermentation-SSF), have been explored to enhance the extraction of bioactives from byproducts and to improve the techno-functional properties of plant-based food byproducts. These pretreatments free and/or activate the bioactives and thus affecting their quali-quantitative composition and by consequence their nutritional quality [57,58,59].

Roasting is a useful thermal treatment that improves the aromatic profile of some food products (coffee beans, green tea, seeds, and rice). Treatment of roasted rice husk at 190 and 230 °C increased the levels of ferulic and vanillic acids and in general resulted in a different chemical profile that was particularly rich in alanine, glutamine, N,N-dimethylglycine, choline, 1,3-dimethylurate, and glucose. Different antioxidant assays confirmed that the best results were achieved from 70% ethanol extracts of rice husks roasted at 230 °C [55].

In recent years, the microbial fermentation of rice bran and germ has been applied to enhance and standardize the bioactive content, which is often closely related to the rice variety. This process can be caused by both endogenous and bacterial enzymes, and the type of fermentation (anaerobic and aerobic) can affect the amounts of compounds released, as well as the generation of potential derivatives, thus influencing the corresponding activity [58]. SSF represents a very promising solution for waste/byproduct recycling because of its capacity to manage solid wastes, maintain biomass energy, and transform wastes into high-value-added products (biofuels, bioplastics, and bioactives). SSF consists of a method resistant to bacterial contamination that uses the solid waste as substrate for bacteria and allows an increase in enzyme efficiency after the careful establishment of different parameters (substrate and substate particle size; microorganism type and inoculum; process conditions, such as temperature, aeration, agitation, solvent type, pH, concentration, solvent/solid ratio, and purification of the final products) and accurate studies required to scale up the process. At present, tray bioreactors represent the easiest method of scaling up SSF, consisting of an aerobic environment maintained by thin perforated trays (plastic or aluminum) with controlled temperature and relative humidity [59]. For example, fungal (*Rhizopus* sp. and *Aspergillus oryzae*) fermentation increases the protein content and antioxidant activity of defatted rice bran [60]. In particular, SSF of rice bran performed with *Rhizopus oryzae* resulted in strong inhibition of peroxidases and polyphenol oxidases, thus increasing the phenolic content and the antioxidant activity of the rice-bran extract [58].

An increase in antioxidant power has been also obtained with yeast fermentation by *Issatchenkia orientalis*, which acts directly on the release of the free polyphenols from bran cell walls. These polyphenols, in turn, act strongly against radical oxygen species (ROS). Conversely, *Rhizopus oligosporus* and *Monascus purpureus* fungi increased the amounts of phenolic compounds, but the different composition of phenolic acids decreased the radical scavenging and antioxidant activities of 2,2-diphenyl-1-picrylhydrazyl (DPPH). The use of both fungi in combination yielded the highest ferulic-acid content and increased ROS inhibition/nitrogen formation and free-radical neutralization [61]. Mixed-bacteria solid-state fermentation seems to be a promising method by which to increase the levels of many antioxidant compounds, such as polyphenols, mainly flavonoids and hydroxycinnamic acids, and proteins. Recently, *Rhizopus oryzae* and *Aspergillus oryzae* or *S. cerevisiae*, *B. subtilis*, and *L. plantarum* mixture have also been successfully used to increase the antioxidant activity of rice-bran extracts [62].

### 3.2. Extraction Procedures: New Solvents and Methodologies

Extraction is the first step in separating compounds from the matrix. Many different methods have been used to extract antioxidant compounds from rice byproducts, including both conventional and nonconvectional extractions. Conventional extraction approaches include solvent extraction (SE) or SE combined with other methods. The conventional techniques commonly used for extracting bioactive compounds from matrices are stirring, Soxhlet extraction, and far-infrared radiation (FIR) [63]. Their principles are very different, with very different results in terms of the content (qualitatively and quantitatively) of the extracted compounds. 

Conventional methods are still used, but as part of the search for eco-friendly and non-toxic solutions, green technologies based on the use of green solvents (mainly water, methanol, ethanol, 1-propanol, 2-propanol, acetone, acetonitrile, and supercritical fluids) have been increasingly explored. These solvents have been chosen because their use saves time and solvent volume and because of their easy management compared to that of organic toxic solvents (e.g., toluene, benzene, chloroform, hexane). In addition, thermolabile compounds cannot be used at the high temperatures generally applied for processing or evaporating organic solvents [64,65].

Among the green solvents, supercritical fluids, mainly supercritical carbon dioxide (SC–CO_2_), are commonly used in supercritical fluid extraction (SFE). They are effective on biomaterials such as herbs and natural plant materials. Supercritical fluids are characterized by temperatures and pressures above their critical values and have gas and liquid properties at the same time, allowing chemical features such as low viscosity, high miscibility, solvating power, and diffusivity. Therefore, these solvents ensure high extraction efficiency and selectivity in short times. CO_2_ is particularly stable and recyclable and has convenient critical temperature (31.1 °C) and pressure values (72.8 bar) that can be obtained easily [66]. CO_2_ is a low-polar solvent suitable for extraction of non-polar compounds such as phytosterols (β-Sitosterol, stigmasterol, campesterol) from rice byproducts. Subcritical water extraction (SWE), which involves water in subcritical fluids with pressure and temperature below the critical points, could also be used, but this technique is generally less efficient than SFE and is specifically useful for extracting water-soluble sugars [67,68,69].

Other green solvents, such as deep eutectic solvents (DESs) and natural deep eutectic solvents (NaDESs) represent a fundamental approach for extracting antioxidants that are safer and environmentally friendlier approaches than traditional extraction solvents [65].

DESs consist of a mixture of Brønsted–Lowry acids and bases that act as hydrogen bond donors (HBD) (e.g., acids, amines, or alcohols) and hydrogen bond acceptors (HBA), (e.g., tetraalkylammonium, quaternary ammonium, or phosphonium salts), respectively. NaDESs are formed by two or three natural molecules (sugars, organic acids, and amino acids), using the same hydrogen interactions as DESs. However, these reactions give rise to a eutectic mixture with a lower melting point than its components and with very good dissolution properties. They are very easy to produce by various processes, such as evaporation, freeze-drying, heating along with mixing, grinding, ultrasound-assisted and microwave-assisted heating. In addition, they are versatile, as the different compositions of HBD and HBA affects all mixture properties (density, viscosity, conductivity, polarity, etc.) [70,71]. 

Fluorous solvents, which consist of high-fluorinated molecules (perfluorohexane, perfluorooctane, perfluoropolyether, and perfluorotributylamine), can be useful to extract organic compounds from food/food byproducts matrices, but they are very expensive and have a few applications, as they are not suitable for highly non-polar solutes [65].

**Figure 2:**
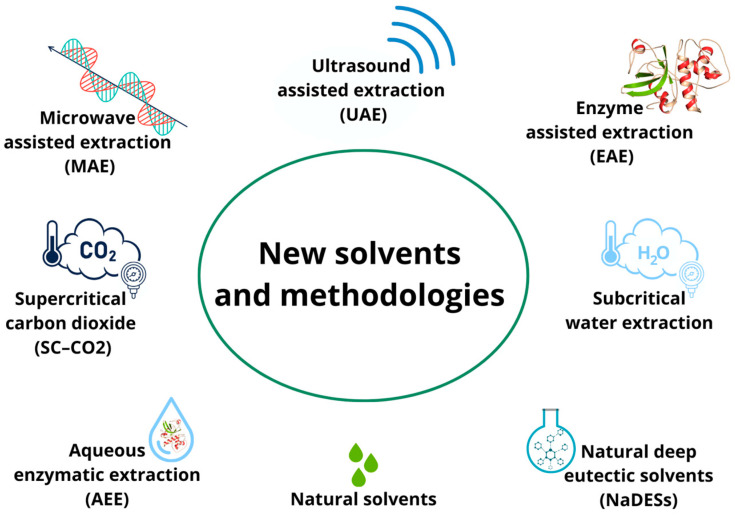
New extraction techniques applied to agri-food byproducts.

In particular, in the past decade, in addition to the search for new solvents, there has been an increasing demand for new extraction techniques that are amenable to automation and have shortened extraction times and reduced organic solvent consumption to prevent pollution and reduce the cost of sample preparation (Figure 2). 

Green extraction techniques have not been deeply investigated for their potential to recover phytochemicals from rice byproducts, but some interesting applications have been developed. In addition to SFE and SWE, microwave-assisted extraction (MAE), ultrasound-assisted extraction (UAE), enzyme-assisted extraction (EAE), and aqueous enzymatic extraction (AEE) are the most-performed green methodologies. These techniques allow the contact between the extracting solvent and the plant matrix to be increased through different principles; this increased contact increases the probability of mass transfer. In more detail, MAE is based on the use of electromagnetic waves that, through high temperatures, excite both the solvent and the matrix, improving efficiency in terms of extraction yield [72]. Similarly, UAE, through the application of ultrasound, generates shear forces, pressure changes, agitation, cavitation, radical formation, and even wave formation, which increase the recovery of bioactive compounds [52]. In contrast, EAE and AEE differ from the previously discussed techniques, as they exploit enzymatic hydrolysis reactions to promote cell-wall lysis and optimize extraction yield. The difference between these two techniques consists only in the presence or absence of water during the extraction process. In AEE, water is used to solubilize the enzyme, and then the enzyme aqueous solution is added to the rice-husk extract [54]. In contrast, in EAE, water is used as an extraction solvent to solubilize the rice husk, and the enzyme is added afterwards [73].

An example showing how different pretreatments/extraction methods can affect the antioxidant activity of the obtained extract is a study performed on rice bran and husk that underwent different pretreatments, non-enzymatic and enzymatic, with hot air (120 °C, 30 min), FIR (energy intensity of 2 kW/m^2^), and cellulase (50 °C, 24 h, pH 5). The highest total phenolic content (TPC), DPPH radical scavenging activity and ferric reducing antioxidant power (FRAP) were obtained for both rice byproducts using FIR. The application of FIR also induced an increase in the amounts of α- and γ-tocopherols extracted from rice bran in comparison to the hot-air treatment. Conversely, cellulase increased the extraction yield for vanillic acid, another polyphenol possessing antioxidant activity [17]. 

For crude RBO, SE, SFE, MAE, and AEE can be easily used, but cold press extraction (CE) is the most promising technique thanks to its low cost and its capacity to extract a high amount of antioxidants, especially when it is combined with thermal processes (thermal-cooking cold press extraction, CCE) or ultrasounds (ultrasound-pretreated cold press extraction, UCE); in addition, more rapid and efficient extractions can be performed using UCE [74]. 

Table 1 summarizes the advantages and disadvantages of different green extraction techniques.

## 4. Antioxidant Activity of Rice Byproducts: Mechanisms of Action and Analytical Techniques for Evaluation

The role of antioxidant compounds is to counteract the formation and the action of reactive oxygen/nitrogen species (ROS/ RNS), which are involved in the pathogenesis and pathophysiology of various chronic diseases, such as neurodegenerative, cardiovascular, inflammatory, diabetes-related, and cancer-related processes [75]. Reactive species like superoxide, peroxide, singlet oxygen, hydroxyl radical, nitric oxide, and peroxynitrite anion are physiologically formed in the mitochondrial respiratory chain following purine nucleotide metabolism, arachidonic acid and prostaglandin synthesis, or enzymatic reaction. They are involved in several systemic processes, such as immune protection or cytotoxicity against pathogens [76]. Normally, endogenous enzymatic systems such as superoxide dismutase and catalase or methionine sulfoxide reductase restrict the activity of reactive species produced in excess, attenuating their damaging effects [76]. However, when ROS production outstrips the antioxidant capacity of defense systems, oxidative stress or nitrosative stress occurs. In such cases, high concentrations of ROS and RNS damage macromolecules like proteins, lipids, and DNA, leading to increased oxidative stress and various human diseases [77]. Therefore, antioxidant compounds can act at different steps of the oxidative radical process, counteracting initiation, propagation, and chain termination. They can also deplete molecular oxygen, decrease its local concentration, chelate prooxidative metal ions, trap reactive species, scavenge chain-initiating radicals, break the chain of a radical sequence, or quench singlet oxygen [78]. Normally, such chain breakers are called primary antioxidants due to their ability to scavenge radical species; they are followed by the secondary antioxidants, which act mainly as singlet oxygen quenchers, metal chelators, and oxidative enzyme inhibitors. Secondary antioxidants may also act in synergy with primary antioxidants, stabilizing them with an acidic environment or regenerating them by hydrogen donation [79].

There are three main categories of methods by which to measure AOA. Spectrometry remains the most used technique, but electrochemical assays and chromatography techniques can also be used. The large number of available assays complicates a comparison of the results. It is a common procedure to perform AOA experiments with different techniques, using a multidisciplinary approach. Through spectrometric methods, the measurement of AOA can be determined mainly by a colorimetric assay (DPPH, 2,2′-azino-bis(3-ethylbenzothiazoline-6-sulfonic acid-ABTS, FRAP) or by measuring a loss of fluorescence of fluorescein (oxygen radical absorbance capacity, or ORAC, test). A limitation of most of these methods is that they measure the intrinsic AOA based on hydrogen-atom transfer or on electron-donating capacity; these results could be very different compared to those obtained in vivo or in a food matrix [80].

The AOA of rice byproducts has been measured mainly by simple, traditional in vitro methods, such as DPPH or ABTS radical scavenging activity assays, FRAP tests, phosphomolybdate assays, and ORAC tests. Antioxidants can act as free radical scavengers, singlet oxygen quenchers, ROS and peroxide inactivators, metal-ion chelators, quenchers of secondary oxidation products, and inhibitors of pro-oxidative enzymes. DPPH, ABTS, and FRAP assays are based on an electron transfer from the antioxidant compound to the radical, which is reduced, resulting in a color change or a discoloration that can be spectrophotometrically monitored. The spectrophotometric measurement of the color change allows the quantification of free radicals in solution and thus of sample AOA levels. Conversely, the ORAC assay monitors the inhibition of peroxyl radical oxidation by measuring the reduction in fluorescein fluorescence [80,81].

Electrochemical techniques based on the use of biosensors and on the application of voltametric or amperometric principles are relatively new, complementary methods to be used in parallel to the traditional tests or, alternatively, when colored or turbid samples complicate spectrophotometric analysis. They use a variation in current generated by sample oxidation/reduction to quantify AOA. The main limitation of these techniques consists of long working times and the need for repeated washing procedures, but in the last few years, some automated assays have been proposed to allow more rapid analysis. Automation thus also allows for screening experiments [80,82]. Recently, a promising amperometric fluidic system has been proposed by Veenuttranon & Nguyen to measure the total AOA as DPPH scavenging activity. This system consists of a pressure-controlled flow and a mixing device. It could be also miniaturized to the microfluidic level, allowing rapid and low-cost measurement of antioxidant activity in food samples [83]. The electrochemical techniques based on voltametric principles are mainly used to measure the antioxidant power of polyphenols, which exhibit low potential when their capacity to donate electrons is high [84].

For complex mixtures, photochemiluminescence (PCL) methods could be an easy and rapid approach by which to measure the integral antioxidant capacity (IAC), which represents the antioxidant capacity of all hydrophilic and lipophilic antioxidants. PCL is based on the photochemical generation of free radicals, as measured using chemiluminescence [85].

It is well known that AOA content is strongly dependent on the types of extraction solvents used and that it can be directly related to TPC and total flavonoid content (TFC), but, often, no correlation between scavenging activity methods and TPC or TFC has been found for rice byproducts [17,86]. Rice bran AOA was extensively investigated by conventional assays such as DPPH and ABTS methods, and the highest TPC and TFC values were obtained using ethanol, rather than other solvents (water, methanol, and acetone). Ethanol was also used to improve TPC and TFC results for husk and germ. Conversely, the highest AOA for rice bran has been found using water and methanol or ethanol, rather than acetone, indicating that solvent polarity generally can positively influence AOA. In summary, ethanol can be considered the ideal solvent through which to simultaneously obtain high extraction yields of phenolic compounds and good antioxidant activity [86].

Only a small number of studies on rice husk and its antioxidant components are present in the literature because rice husk is not an edible food matrix, as mentioned above. As for rice bran, ethanol is the ideal solvent for the recovery of polyphenols from husk. In particular, Soxhlet or ultrasonic-bath extractions performed with 75% ethanol under magnetic stirring have been performed with and without an initial grinding step (mortar and pestle). The results of the comparison show that grinding (around 60–75 min) widely increased the total phenol yields. Lignocellulosic materials (cellulose, hemicellulose, and lignin) from rice husk can be pretreated with an acidic (2% sulphuric acid, 90 °C) or basic (2N NaOH, 150 rpm) hydrolysis to improve the recovery of antioxidant compounds. The use of basic conditions resulted in the best total phenol yields in 90–120 min and in the extract with the highest antioxidant power (measured by phosphomolybdate, FRAP, and DPPH assays). In addition, the extracts obtained under these conditions had the highest superoxide radical scavenging activity, hydrogen peroxide scavenging activity, and hydroxyl radical scavenging activity, although these values were not significatively different from those obtained under acidic conditions [87].

## 5. Examples of Green Methodologies Applied in the Recovery of Antioxidant Compounds from Rice Byproducts 

### 5.1. Rice Husk

Green extraction techniques have not been deeply investigated for the recovery of phytochemicals from rice husk. Nonetheless, in the last ten years, some green applications using husk have been reported in the literature. For example, Jiamphun et al. exploited the EAE advantage of reduced time and cost to increase the extraction yield of antioxidant compounds from glutinous rice husk [54]. It is known that glutinous rice husk contains a higher content of antioxidants than does non-glutinous rice husk [88]. The aim of this study was to verify the yield and the quality of vanillic and ferulic acids, as well as their antioxidant activity. Briefly, the authors compared a traditional extraction by maceration with 95% ethanol (*v*/*v*) for 3 days with AEE. The optimal conditions used for AEE were glutinous rice husk/solvent ratio 1:5 (*w*/*w*), 0.5% cellulase aqueous solution, pH 6.5, 50 °C, and 24 h. The study found that AEE enhanced the extraction efficiency of vanillic and ferulic acids; the vanillic acid content was 10.9 ± 0.9 mg/g of extract with AEE, which was significantly higher than the concentration obtained using ethanol (4.5 ± 1.9 mg per gram of extract) (*p* < 0.05). Additionally, ferulic acid was detected only when AEE was used, probably due to plant cell-wall degradation induced by the cellulolytic effect of cellulase and to the slightly acidic pH (6.5) of the cellulase enzyme aqueous solution. An acidic solution was probably able to ionize the phenolic compounds, making them more soluble. Regarding AOA, AEE significantly increased radical scavenging activities against DPPH and ABTS radicals (IC50 DPPH: 201.3 ± 13.7 µg/mL; TEAC: 6.5 ± 0.3 µg Trolox/mg extract) compared to traditional EtOH extraction (IC50 DPPH: 184.0 ± 20.8 µg/mL; TEAC: 0.0 ± 0.8 µg Trolox/mg extract). Jiamphun et al., Park C.Y. et al., combined AEE with high-hydrostatic-pressure (HPP) extraction to recovery tricin, a flavonoid with antioxidant properties [54,89]. The authors initially evaluated the effect of the extraction techniques separately, but the combination of the two approaches resulted in excellent yields of tricin (32.9 mg/kg rice hull) and other phytocompounds. The main reason for the difference could be the easier extraction of intercellular bioactive compounds from plant-based matrices with AEE, while HPP shortened the extraction time and increased the solvent permeability.

The synergy of the two techniques also increased the ABTS radical scavenging activity of a rice-husk extract by about 20% compared with the control extracted by a traditional method. Therefore, the use of EAE in combination with other ecofriendly extraction techniques is a winning strategy, as also demonstrated by Vardakas et al. in their study [90]. They used EAE in combination with SWE in order to recover polyphenols from rice husk. The first step consisted of the use of SWE in a static mode at 160 °C for 60 min using a solid/solvent ratio of 1:11 (*w*/*v*) to produce rice-husk extract. The second step consisted of the addition of 20% *w*/*w* enzymatic mixture solution (cellulase:pectinase:xylanase = 3:1:1) to the solid residue obtained from the first step. Another SWE step was performed on the solid residue after 7 h of incubation at 40 °C. The TPC content obtained from the combination of the two extraction techniques was 2.97 g gallic acid equivalent (GAE)/kg rice husk and was very similar to that obtained by traditional hydroalcoholic extraction (ethanol 70%). This high TPC value probably resulted from both solvent polarity and enzymatic hydrolysis, which facilitate the degradation of the plant wall and enhance recovery of polyphenols. The need for polar solvents was also confirmed by Kaur et al. in the extraction of high-value phenolic compounds from rice husk using ethanol and water modified with supercritical carbon dioxide (1.29 mg GAE/g of TPC and 0.40 mg catechin equivalent (CE)/g of TFC) [91]. SC–CO_2_ extraction is considered a safe and environmentally friendly method for extracting bioactive compounds; however, SC–CO_2_ alone has a limited ability to extract phenolic compounds due to their polar nature [92]. Conversely, an ethanol–water mixture used as polar co-solvents enhanced the solubility of polyphenols, thus improving their mass transport. Moreover, the use of ethanol–water-modified SC–CO_2_ improves the solvating power, hence increasing AOA (0.23 mg TE/g).

Another green solution used by Jha et al. contributed to the evidence supporting a positive effect of co-application of MAE and UAE on the extraction of antioxidant polyphenols from black rice husk [93]. The optimal conditions, according to their experiment, were sonication for 10.02 min at 49.46 °C, solute/solvent ratio 1:40.79 (*w*/*v*), 67.34% ethanol in the extraction solvent, and microwave application for 31.11 sec. This innovative extraction method reduced extraction time and cost, increasing recovery yields of phenolics (1.72 mg/g GAE), flavonoids (3.01 mg/100 g), and anthocyanins (3.36 mg/100 g) because the penetration of the extraction solvents into the plant matrix is promoted by the action of electromagnetic waves and ultrasound.

### 5.2. Rice Bran

The antioxidant compounds in rice bran and RBO have also been recently extracted using green approaches. For example, Kaewchuen et al. investigated the use of SWE under optimized conditions, i.e., ethanol 95% *v*/*v*, sample/solvent ratio 1:5, and incubation at 200 °C for 30 min [94]. An increase in TPC yield (62.72 mg GAE/g) was found in comparison with the results obtained by applying two Soxhlet methods using hexane and ethanol 95%, respectively (hexane 0.22 mg GAE/g and ethanol 4.52 mg GAE/g). The same authors also applied SSF using *Aspergillus* oryzae on defatted rice bran and optimizing the experimental conditions (*A. oryzae* 6.8 × 10^8^ spores/g material, fermentation time 7 days, 65% RH, pH 5.5). SSF is widely used as a pretreatment to improve the antioxidant properties of rice bran [62]. Rice bran, unfermented defatted rice bran, fermented defatted rice bran, and a mixture of fermented and unfermented defatted rice bran samples were also extracted using SWE and TPCs, and the results were compared. SSF applied to defatted rice bran increased TPC from 10.23 to 35.56 g GAE/g dry matter. A higher TPC value in was also found for the fermented mixture (98.28 mg GAE/g dry matter) using this approach.

UAE with ethanol and D-limonene as solvents was used to extract antioxidants from riceberry bran [44]. The purpose was to compare UAE with a conventional SE and investigate how the polarity of the solvent affects the extraction yield. The extraction yield was higher with UAE than with SE for both solvents (46.58% and 19.69% with UAE using ethanol and D-limonene, respectively, vs. 34.20% and 14.70% with SE using ethanol and D-limonene, respectively). It is notable that UAE gave better yields than SE due to the “sponge effect”, which predicts the growth of cavitation bubbles by ultrasound irradiation and facilitates solvent entry and subsequent mass transfer. Additionally, the effect of ethanol on the extraction yield of highly polar antioxidants (phenolic acids, flavonoids, anthocyanins, and vitamin E) was better than that of D-limonene, which was optimal for extracting γ-oryzanol. Additionally, they found that riceberry bran extracted with ethanol by UAE had the strongest AOA according to a FRAP assay (110.73 mmol Trolox/g) and DPPH radical scavenging activity (14.47 mmol Trolox/g). This study confirmed the utility of UAE for the extraction of antioxidant compounds from this matrix.

The use of new natural solvents (NADEs) coupled with UAE was investigated to extract ferulic acid from defatted rice bran [95]. The application of UAE-NADES (50 W, 50 °C, 50% duty cycle, and 16 min) only slightly improved extraction compared to the use of NADEs alone (9.34 mg/g vs. 8.71 mg/g), but significantly increased the yield compared with SE (9.34 mg/g vs. 3.22 mg/g).

Good results have also been obtained using EAE (35 °C, pH 3.0, 4 h) coupled with enzymatic hydrolysis (1.0% cellulase) [73]. Hydrolysis altered the cell walls of the rice bran, facilitating the recovery of antioxidants (14.3 ± 2.2 mg ferulic acid/100 g vs. 3.45 ± 0.9 mg ferulic acid/100 g obtained without enzyme). However, AOA levels in the samples extracted with and without the enzyme were not very different, as measured by the ABTS assay.

Enzymatic hydrolysis has also been applied by Xu et al. to study antioxidant compounds and activity in RBO [96]. An AEE-extracted oil (AEEO) was obtained with a 1:7.5 matrix/water ratio (*w*/*v*), pH 9.0, and the addition of 2% (*w*/*w*) alcalase before incubation at 57 °C for 150 min. The usefulness of this green extraction was evaluated by comparing it with a conventional Soxhlet oil extraction (SEO), considering total tocopherols and tocotrienols content and AOA as measured by DPPH assay. AEEO yielded a significantly higher (*p* < 0.05) concentration of total tocopherols and tocotrienols (1004 mg/kg) compared with SEO (839 mg/kg). This difference was also seen in the ability to scavenge DPPH free radicals (EC50 5.52 ± 0.10 mg/mL vs. 8.66 ± 0.31 mg/mL).

Another green extraction technique used for the valorization of RBO was SC–CO_2_ technology. Temperature (40 and 60 °C), pressure (30 and 40 MPa), and the amount of ethanol used for the extraction of polyphenols, flavonoids, γ-oryzanols, and tocopherols (0, 5 and 10%) have been investigated [97]. The use of ethanol as a cosolvent improved the extraction yield in terms of TPC (3.42 ± 0.12 mg GAE/g at 30 MPa, 40 °C, and 10% ethanol), TFC (4.47 ± 0.30 mg QE/g of oil at 40 MPa, 40 °C, and 10% ethanol), γ-oryzanol content (20.63 ± 0.64 mg/g of oil at 40 MPa, 40 °C, and 5% ethanol), and tocopherols (5.47 ± 0.30 mg/g of oil at 30 MPa, 40 °C, and 10% ethanol). This co-solvent (10%) also allowed the recovery of bioactive compounds with higher antioxidant activity (DPPH IC50 0.44 mg/mL) at 30 MPa and 60 °C than was seen in compounds extracted with traditional methods using hexane (DPPH IC50 1.02 ± 0.02 mg/mL).

Similarly, SC–CO_2_ combined with fraction extraction was used to assess the content of tocols and γ-oryzanol [98]; the highest concentration was obtained at 41.4 MPa, as it was for Benito-Román et al. [97] in a previous work. Pressure strongly affects SC–CO_2_, as reported by Pinto et al. In fact, in a study using rice bran obtained from different Portuguese varieties (*Japonica* varieties such as *Aries*, *Euro*, and *Opal* and *Indica* varieties such as *Minima*, *Ellebi*, *Sprint*, *Gladio*, and *Sirius*), the best extraction yield, antioxidant activity, and fatty-acid content were obtained with a pressure of 40 MPa (as well as a CO_2_ flow rate of 1 L/min and loading 20 g of rice bran) [99]. As an alternative to SFE, soybean oil was used as natural solvent [100]. The choice of oil as an extractant allowed the use of green techniques and provided a barrier to prevent oxidation and degradation of the samples. In this case, the extraction method was an ultrasound-assisted soybean-oil extraction (O-UASO). The optimal conditions were set to 40% amplitude and 65 °C solution temperature for 40 min. No significant difference was found in terms of the recovery of tocols and γ-oryzanol between O-UASO and a conventional solvent extraction (O-CSE), which was used as a control, however AOA was much higher with O-UASO (2.69 ± 0.03 mg GAE/g oil), probably because the use oil made it possible to also recover thermo-sensitive lipophilic bioactives (e.g., phenolic acids and carotenoids), which degrade during conventional extraction procedures. In addition, Garofalo’s work showed that the UAE technique is less environmentally impactful than a conventional technique. That study compared AUE with a conventional extraction, both using propane as solvent, to obtain rice bran oil. Although the two techniques resulted in similar recovery yields, the extraction time was significantly lower using UAE (1 min) than using the conventional method (15 min) [101].

As evident from the results here reported for rice husk and bran, the use of green technologies generally improves the extraction efficiency and by consequence the extract antioxidant activity in comparison to the use of traditional organic solvents. In addition, the combination of two techniques, such as AEE and HPP, EAE and SWE, and MAE or NADES and UAE, speeds up the extraction process, usually resulting in a TPC content higher than or at least comparable to that obtained by traditional methods. AEE and EAE can significantly improve the antioxidant activity of extracts by increasing polyphenol solubility through strong action on plant cell walls, thus facilitating the recovery of these compounds. In the SC–CO_2_ technique, temperature (40–60 °C) and especially pressure (30–40 MPa) are fundamental parameters whose optimal settings vary for different compounds. A pressure value of 40 MPa generally strongly increases recovery of flavonoids, tocopherols, and γ-oryzanols and therefore increases antioxidant activity in the extract.

Table 2 and Table 3 summarize the results for antioxidant activity (DPPH assay) and the phenolic content (TPC), as obtained by applying different extraction methods to rice byproducts.

## 6. Antioxidant Activity of Rice Byproducts: Relationship between Structure and Antioxidant Properties

This section focuses on the possible mechanism by which the compounds identified in the rice byproducts, mainly phenolic acids, flavonoids, γ-oryzanol, and E vitamins, exert their AOA, elucidating SAR.

### 6.1. Phenolic Acids

The AOA of phenolic compounds is correlated to the ability to scavenge reactive radical species, transferring their electron and/or hydrogen atom to the radical by different chemical pathways. This reaction involves the proton-coupled electron transfer (PCET) reactions, which consist of redox processes with one or more reactants/kinetic steps and different types of transfer: consecutive transfer, referred to as electron transfer/proton transfer (ET/PT) or concerted transfer, the so called ETPT mechanism [102].

However, the chemical structures of phenolic acids affect their antioxidant activity because this activity depends on the presence, number, and arrangements of hydroxyl groups in ring structures, on the electron-withdrawing properties of the carboxylate group in benzoic acid, and on the presence of a methoxyl group [102]. Table 4 shows the chemical features of the phenolic-acid derivatives identified in rice byproducts.

Moazzen et al. compared the AOA of several hydroxybenzoic and hydroxycinnamic derivates previously identified in rice byproducts, such as 4-hydroxybenzoic acid, gallic acid, vanillic acid, syringic acid, ferulic acid, caffeic acid, and cinnamic acid, evaluated as anti-DPPH activity [103]. Based on the results, gallic acid showed better activity than 4-hydroxybenzoic acid due to the high number of hydroxyl groups, highlighting a strong correlation between the presence of more than one hydroxyl group and antioxidant properties. The hydroxyl group promotes the formation of a phenoxyl radical to complete the free-radical chain reaction and can also stabilize the phenoxyl radical by electron donation. Similarly, methoxyl groups in the *meta* position contribute to the antioxidant activities of vanillic acid and syringic acid, stabilizing the unpaired electron of the phenoxyl radical by acting as electron donors. These results are supported by the poor activity of cinnamic acid, which lacks hydroxyl substituents on the aromatic ring. In addition, the catechol moiety stabilizes the formed phenoxyl radical through intramolecular hydrogen bonding, enhancing its antioxidant activity, as shown by the increased activity of caffeic acid compared to ferulic acid.

Recently, the results obtained from DPPH, ABTS, and FRAP assays have been supported by computational methods based on density functional theory (DFT), which is particularly useful to supporting better understanding of the experimental results and predicting antioxidant properties. These methods focus on thermodynamic parameters that affect the radical-scavenger activity, such as phenolic O-H bond dissociation enthalpy (BDE), ionization potential (IP), proton dissociation enthalpy (PDE), proton affinity (PA), and electron transfer enthalpy (ETE), all of which are related to the different PCET pathways cited above [104]. For example, Chen et al. evaluated the structure-antioxidant activity relationship of methoxy, phenolic hydroxyl, and carboxylic acid groups of 4-hydroxybenzoic acid, vanillic acid, syringic acid, protocatechuic acid, ferulic acid, *p*-coumaric acid, isoferulic acid and sinapic acid, combining experimental results with computational studies [105]. Based on the results obtained from DPPH assays, hydroxycinnamic derivates (*p*-coumaric acid, caffeic acid, ferulic acid, isoferulic acid, and sinapic acid) appeared more active than benzoic-acid derivatives (4-hydroxybenzoic acid, vanillic acid, syringic acid, protocateutic acid) due to the presence of a -CH=CHCOOH group, which promotes free-radical scavenging, increasing the electron-cloud density of the aromatic ring and decreasing the dissociation energy of O-H. The low value of O-H BDE calculated for hydroxycinnamic derivates confirm the positive influence of the acid group on the electron-transfer ability of OH groups. Similarly, the introduction of a methoxy group has important effects on AOA, as it reduces the BDE value of the phenolic hydroxyl group, which facilitates breaking of the O-H bond and decreases PA and ETE values. BDA, PA, and ETE values are correlated to an increase in the electron-donating ability of the phenolic acid, according to the greater activity of syringic and synapic acids in comparison to isoferulic and 4-hidroxybenzoic acids, as observed in DPPH assays. In addition, a strong correlation between the position of the hydroxyl group on the aromatic ring and antioxidant activity has been observed by Spiegel et al. [106]. The authors investigated the antioxidant activity of different phenolic-acid compounds using a FRAP test combined with a theoretical DFT approach. SAR investigation showed that compounds with more hydroxyl groups in *orto* and *para* position to each other, such as gallic, caffeic, and protocatechuic acids, exhibited the highest efficacy as antioxidants. Indeed, when a second hydroxyl group is present in the near position or on the opposite side of the ring, it allows a second oxygen atom to stabilize the radical by delocalization. Similarly, the hydroxyl group in the *para* position of ferulic acid positively affects its antioxidant properties, as ferulic acid is more active in DPPH and FRAP than is isoferulic acid, which is *meta*-substituted [107]. In addition, the planar structure of ferulic acid increases its capacity to neutralize free radicals because it promotes π-electron delocalization in the phenyl ring, vinyl bond, and carboxyl moiety, stabilizing the structure [108]. Therefore, ferulic acid, which is the most representative compound of rice byproducts, showed important antioxidant structural properties. This result was supported by the low value of OH-deprotonation energy of ferulic acid in water, which makes it an excellent chelating agent [108]. In addition, the ester derivatives of ferulic acid, such as 24-methylenecycloartanyl ferulate, cycloartanyl ferulate, campesteryl ferulate, and β-sitosteryl ferulate are the main components of γ-oryzanol, which has been identified in rice bran and bran oil [49]. It is clear that the antioxidant activity of γ-oryzanol is due in part to the hydroxyl group and carboxyl moiety of the ferulate portion and in part to the sterolic portion, which is responsible for the higher activity in the lipid environment and thus the ability to counteract lipid peroxidation [109]. In fact, ferulate derivatives effectively inhibit cholesterol oxidation due to their structural similarity. However, the presence of a methylene group on C24 increases the antioxidant activity of 24-methylenecycloartanyl ferulate to a level above that of other components [110].

**Figure 3:**
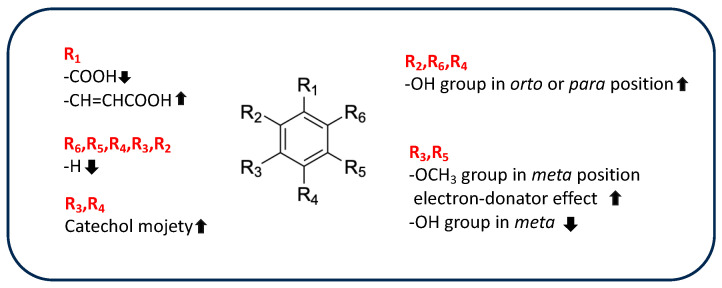
Influence of different substituents of the phenolic acid moiety on antioxidant activity.

Computational evaluation of the antioxidant potential of chlorogenic acid revealed that, despite having many hydroxyl groups, its antioxidant activity is mainly due to the hydroxyl group in position 1 on the aromatic ring. In fact, the lower BDE value of 1-OH is related to a high hydrogen-donating capacity; the formed free radical is stabilized by four intramolecular hydrogen bonds and by delocalization of unpaired electrons in aromatic ring A. In addition, based on BDE value, chlorogenic acid has an antioxidant potential greater than that of other phenolic acids, such as vanillic, sinapic, syringic, *p*-coumaric, and 4-hydroxybenzoic acids [111]. Finally, SAR for radical scavenging properties of hydroxybenzaldehyde has been investigated in vitro (by DPPH and ABTS) by Bountagkidou et al. [112]. It is interesting to note that protocatechuic aldehyde showed similar or sometimes greater antioxidant activity than the corresponding acid derivative in all assays performed, highlighting the greater influence of a catechol structure compared to the carbonyl group. In contrast, other aldehyde derivatives, such as 4-hydroxybenzaldehylde, exhibit less activity than their acid derivatives because of the absence of the acid group. A summary of the different phenolic-acid substituents involved in AOA and their effects on activity is shown in Figure 3.

### 6.2. Flavonoids

Flavonoids are a group of hydroxylated phenolic compounds that are differently substituted, well known for their antioxidant properties, and characterized by a common skeleton of phenyl-benzo-gamma-pyran (C6-C3-C6). This skeleton, also known as the flavan nucleus, is composed of two phenyl rings (A and B) connected through a pyran ring (C) [113]. As for phenolic acids, flavonoids can transfer hydrogens or electrons to free radicals by different chemical pathways and can also act as chelating agents. However, the chemical structure of a given flavonoid affects its antioxidant activity, as there is a correlation between radical scavenging activity and the presence of the catechol group at position C3′ and C4′ on ring B, the double bond at position C2=C3 conjugated with the 4-oxo function in C4, and hydroxyl groups in positions C3 [113]. Table 5 shows the different flavonoid chemical features that contribute to the AOA of rice byproducts.

**Figure 4:**
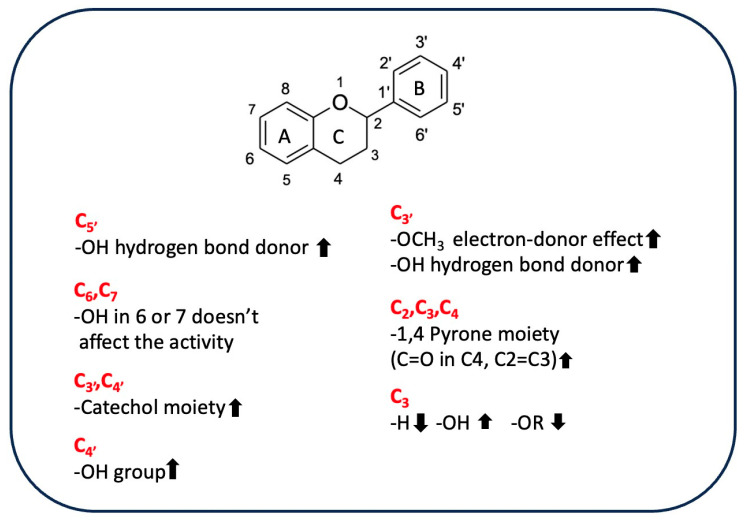
Influence of substituents on the antioxidant activity of flavonoids.

A study performed by Ahmadi et al. has shown how catechin was more efficient than luteolin in reducing ferric ion (Fe^3+^) to ferrous ion (Fe^2+^) in a FRAP test due to the greater electron-donor effect exerted by the 3–OH group [114]. The structure of luteolin is characterized by a 1,4-pyrone moiety; this moiety promotes π-electron delocalization from the B ring, which is also enhanced by the planar structure and resonance effects. Nevertheless, luteolin exhibits higher ionization potential. This potential is related to electron-donating capacity, evidencing a greater instability of the formed phenoxyl radical than is seen with catechin, in which the 1,4-pyrone moiety is lacking and the saturated ring is substituted by a hydroxyl group in C3. Therefore, the 3–OH group has the greatest effect on antioxidant activity, efficiently transferring a single electron to the electron-accepting radicals. However, the concomitant presence of the 1,4-pyrone moiety and the 3–OH group enhances the antioxidant properties of quercetin, which is the most effective free radical scavenger due to its C2=C3 double bond conjugated with the aromatic ring, which promotes the monoelectronic oxidation of the hydroxyl group linked to the C3 [115], as was also found by Sroka et al. [116]. A comparison of the antioxidant activity of selected flavones, such as luteolin and apigenin, and flavonols, such as quercetin, myricetin, and apigenin, showed that hydroxyl group and its position significantly influence antioxidant properties, as the main difference between the compounds belonging to the two classes is the presence/absence of the -OH group in C3. Based on the experimental results using DPPH, FRAP, and ABTS assays, flavonols are more active than flavones due to the presence of the 3-OH group. The exception is luteolin, which is the most active flavone despite the lack of a 3-OH group because of the catechol moiety in C3′–C4′ on ring B, which positively affects its antiradical activity. The formation of a diradical and the rearrangement of a catechol moiety to 1,2-benzoquinone are the most widely used explanations for the difference in antioxidative scavenging potential between luteolin and apigenin [117]. These results are supported by the lower minimal hydrogen abstraction calculated with DFT, which showed that the 4′-OH, 3′-OH, and 3-OH groups are strongly correlated with the antioxidant properties of flavonoids, as revealed by the increasing activity of quercetin, myricetin, and luteolin [116]. The removal of hydrogen from the 3–OH group allows the molecule to achieve an exactly planar structure, increasing the delocalization of free electrons located on oxygen atoms connected with the aromatic ring. These electrons then interact with the orbitals of carbon atoms in conjugation and hyperconjugation effects, decreasing the system’s total energy. On the other hand, if there are more -OH groups on the B-ring, C4′ will be selected due to the possibility of establishing hydrogen bonding with the hydroxyl residues adjacent to C3′ or C5′. For example, in myricetin, the hydrogen-atom transfer (HAT) mechanism (concerted transfer of a proton and an electron in a single kinetic step) seems to be favored because C4’ has the lowest value of BDE [117]. Also, tricin, which has three O−H bonds at the C5, C7, and C4′ positions involved in the formation of phenoxide radicals, the most stable radical is formed by the cleavage of the C4′ O−H bond in the B ring, as this bond has the lowest BDE. However, the tricin phenoxide radical formed is less stable than the myricetin radical derivative because the methoxyl groups in positions C3′ and C5′ do not stabilize the formed radical via hydrogen bonds. Based on the results of a DPPH assay and DFT studies, myricetin is a better radical scavenger than tricin. In general, these data show that the nearby hydrogen bond/s stabilize a radical more than the planar structure does [118]. Moreover, the presence of a sugar moiety linked to the 3-OH group reduces the antioxidant activity of rutin compared to that of quercetin in DPPH and ABTS systems [115]. Conversely, electrochemical and quantum-mechanical analysis of the antioxidant activity of 6-hydroxylflavone and 7-hydroxylflavone showed that a hydroxyl group in C6 or C7 position on the ring A did not significantly affect the ability to scavenge free radicals due to the site’s low susceptibility to oxidation, making these molecules similar to the compounds without -OH groups in positions C6 and C7 [84]. In addition, quercetin, rutin, and catechin had excellent chelating properties toward iron due to the catechol moiety, which is involved in the interaction with metal [119]. In a different context, the antioxidant properties of anthocyanins are affected by structural features such as hydroxylation and methoxylation of B ring and the type, site, or number of glycosylations [120,121]. In fact, the presence of the sugar moiety is responsible for the reduction in the antioxidant activity of the peonidin-3-*O*-glucoside respect to peonidin aglycon because the molecule lacks one of the hydroxyl groups. This difference affects the structure and planarity of the molecule by changing its conjugation capacity and thus reduces its chelating properties [122]. Conversely, it was found that the antioxidant activity of cyanidin did not differ significantly from that of its 3-*O*-monoglycosides derivatives, at least in a system composed of a 1-palmitoyl-2-oleoylphosphatidylcholine (POPC) lipid bilayer, in which lipid peroxidation was induced by 2,2′-azobis (2-amidinopropane) dihydrochloride (AAPH) and hydrogen peroxide (H_2_O_2_) was used. By contrast, the presence of a disaccharide linked to the aglycone reduces the antioxidant activity of this compound because it induces a structural change, making the OH groups responsible for AAPH-induced free-radical scavenging less available. However, in the presence of H_2_O_2_, which is an oxidizing agent, no differences were observed in the activity of the two molecules, highlighting a connection between the activity of the tested compound, the type of radical, and the test used [123]. Figure 4 shows substituents of flavonoids and their effects on antioxidant activity.

### 6.3. Vitamin E

As evident from the phytochemical analysis of rice byproducts, tocopherols and tocotrienols were identified in all isoforms (α, δ, γ, β). Their chemical structure is composed of a chromanol ring with a hydroxyl group at C6, differently substituted with methyl groups (according to the isoform type), a saturated phytolytic side chain for tocopherols, and an unsaturated side chain with three double bonds at the 3′, 7′, and 11′ positions for tocotrienols [124]. The antioxidant properties of E vitamins are correlated with the presence of the phenolic hydroxyl group at the C6 position, although the various isoforms exhibit different antioxidant capacities that seem to be influenced by the methyl groups and, rarely, by the side chain. In addition, several studies have also reported a strong influence of the method used for evaluation of antioxidant properties [125]. Table 6 shows the different isoforms of tocopherols and tocotrienols identified in rice byproducts.

**Figure 5:**
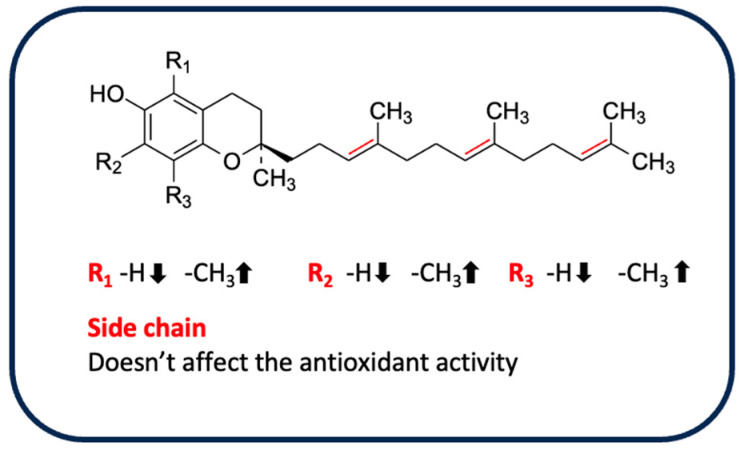
Influence of substituents on the antioxidant activity of tocopherols and tocotrienols.

For example, Bakhouche et al. evaluated the influence of substituents on the mechanism of antioxidant action of α, δ, γ, β-tocopherols in gas phase through theoretical study (DFT method), using as a model a chromanol (TOH), in which the side chain is replaced with an H-group with a small influence on the antioxidant properties [126]. The results indicated that the methyl group, mainly in the *ortho* position, exerts an inductive and hyperconjugation effect, promoting electron delocalization and increasing the stability of the tocopheroxyl radical. When α-TOH is substituted with three methyl groups, it becomes more active than the other isoforms β, γ and δ, which have no group in *ortho* or only one, respectively. This difference could be due to the lower values of O–H bond dissociation free energy, ionization potential, and proton affinity compared with the other forms in gas phase [126]. Similarly, the lipophilic antioxidant activity of tocopherols and tocotrienols, as evaluated by a PCL method, confirms the increased activity of α-tocopherol and α-tocotrienol, followed by the β, γ and δ isoforms, emphasizing once again a close correlation between antioxidant properties and the degree of methylation in the *ortho* position of the chromanol ring. Despite the small influence of the side chain on antioxidant activity, higher activity was observed in the corresponding unsaturated derivative only for the α-isomer [127]. As already anticipated, the conditions and the oxidizing agent used affect the radical scavenging capacity. Muller et al. evaluated the antioxidant properties of vitamin E isomers by several in vitro assays (DPPH, ABTS, FRAP, and ORAC) [128]. In almost all assays performed, side chains were not observed to influence the activity of tocopherols and tocotrienols. The exception was the ORAC test, in which α-tocotrienol was more active than the other isoforms, a result in agreement with what Karmowski et al. reported [127]. By contrast, a strong effect of the degree of methylation was observed only in the FRAP test (with the highest activity attributed to the α-isomer). However, the ORAC test indicated a trend in activity: isomers without methyl groups in 7, such as β and δ, were the most active, probably due to steric hindrance [128]. Overall, the α-isomers appear to be the most active. These isomers are known as chain-breakers as a result of the strong stability of the phenoxyl radical, which is due to stereoelectronic and electron-donor effects exerted by the 2p-type ion pair of electrons of the heterocyclic oxygen atom in the *para* position to the phenolic group and the methyl group on the ring, respectively [125]. A summary of the influence of different substituents on antioxidant activity is shown in Figure 5.

## 7. Conclusions

The valorization of agri-food byproducts as a natural and economic source of compounds is a very important strategy for sustainable development. The implementation of technologies to improve fermentation procedures and green methodologies in terms of high efficiency and yields are fundamental to obtaining cost-effective natural molecules and products for developing food or cosmetic ingredients. Currently, rice consumption is increasing worldwide; by consequence, the management of rice byproducts is a global issue. In particular, rice bran and germ contain high amounts of bioactives with antioxidant activity (phenolic acids, flavonoids, and vitamin E). The use of microbial fermentations, such as solid-state fermentation, represents a promising scalable solution for obtaining high-value-added products with high antioxidant activity at the industrial level. Such green technologies are perfectly suited to the concept of complete and sustainable recovery of byproducts. Among green extraction techniques, supercritical fluids, microwaves, ultrasounds, and enzymes have been recently attempted, mainly for rice bran, with interesting results in terms of the recovered amounts of bioactives. They thus represent a promising strategy for industrial applications. In addition, the establishment of advanced analytical procedures could allow researchers to identify and quantify the components of byproducts and accordingly identify their potential activities and uses.

## Figures and Tables

**Table 1:** Advantages and disadvantages of different green extraction techniques.

Green Extraction Technique	Advantages	Disadvantages
Supercritical fluid extraction (SFE) with SC–CO_2_	rapidity high efficiency good selectivity versatility mild extraction suitable for thermolabile/volatile compounds suitable for non-polar compounds	medium costs not suitable for polar compounds limited mass transfer coextraction of undesirable substances
Subcritical water extraction (SWE)	tunable selectivity suitable for thermolabile/volatile compounds suitable for water-soluble compounds preservation of compounds activity	medium efficiency large extract volumes and low concentration in final products complex optimization often, long times; potential formation of undesirable substances
Deep eutectic solvents (DES) extraction	high dissolution properties easy production stability low costs versatility	high viscosity toxicity of some DESs
Fluorous solvents extraction	rapidity stability versatility suitable for polar compounds	high costs not suitable for highly non-polar compounds
Microwave-assisted extraction (MAE)	rapidity high efficiency high precision homogeneous temperature diffusion mild extraction	highly dependent on solvent/biomass dielectric properties often, long times; potential formation of degradation products complex microwave-system selection difficult scale-up
Ultrasound-assisted extraction (UAE)	rapidity high efficiency	non-uniformity in ultrasonic-energy distribution potential formation of degradation products
Enzyme-assisted extraction (EAE)	rapidity high efficiency high selectivity non-necessity of other processing steps	high costs complex optimization difficult scale-up
Aqueous enzymatic extraction (AEE)	rapidity versatility mild extraction preservation of compounds’ activities stable products	medium efficiency high costs long times emulsification of products and necessity of other processing steps
Cold press extraction (CE)	high efficiency low costs	necessity of other processing steps

**Table 2:** Summary of extraction methods applied to rice byproducts and antioxidant activity of the extracts, expressed as DPPH antiradical activity.

Rice Byproduct		Extraction Method	DPPH	Reference
Husk		AEE	201.3 ± 13.7 µg/mL	[88]
		EAE-HPP	n.d.	[89]
		SC–CO_2_	n.d.	[91]
Bran	Pigmented Not pigmented	UAE EAE	14.47 mmol Trolox/g 2.94 mM Trolox	[44] [73]
Bran oil		AEEO	5.52 ± 0.10 mg/mL	[96]
		SC–CO_2_	0.44 mg/mL	[97]
		O-UASO	215.6 ± 11.2 IC50, mg/g	[100]

**Table 3:** Summary of extraction methods applied to rice byproducts and total phenolic content of the extracts.

Rice Byproduct	Extraction Method	TPC	Reference
Husk	EAE + HPP	3.299 mg GAE/kg of RH	[89]
	SC–CO_2_	1.29 mg GAE/g of RH	[91]
	MAE + UAE	1.72 mg GAE/g of RH	[93]
	AEE	180.0 ± 1.7 mg GAE/g of RH extract	[54]
	EAE + SWE	2.97 mg GAE/g of RH	[90]
Bran	SWE + SSF	98.28 mg GAE/g of RB not pigmented	[94]
	UAE	109.71 mg GAE/g of RB pigmented	[44]
Bran oil	SC–CO_2_	3.42 ± 0.12 mg GAE/g of RBO	[97]
	SC–CO_2_	3.07 mg GAE/g of RBO	[99]

**Table 4:**
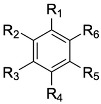
Comparison of different substituents of phenolic acids and derivatives.

Compound	R_1_	R_2_	R_3_	R_4_	R_5_	R_6_
Ferulic acid	CH=CHCOOH	H	OCH3	OH	H	H
Isoferulic acid	CH=CHCOOH	H	OH	OCH_3_	H	H
*p*-Coumaric acid	CH=CHCOOH	H	H	OH	H	H
Protocatechuic acid	COOH	H	H	OH	OH	H
Syringic acid	COOH	H	OCH_3_	OH	OCH_3_	H
Cinnamic acid	CH=CHCOOH	H	H	H	H	H
Vanillic acid	COOH	H	H	OH	OCH_3_	H
Sinapic acid	CH=CHCOOH	H	OCH_3_	OH	OCH_3_	H
4-Hydroxybenzoic acid	COOH	H	H	OH	H	H
Caffeic acid	CH=CHCOOH	H	OH	OH	H	H
Chlorogenic acid	CH=CHCOOR ^a^	H	OH	OH	H	H
Gallic acid	COOH	H	OH	OH	OH	H
Protocatechualdehyde	COOH	H	H	OH	OH	H
4-hydroxybenzaldehyde	COOH	H	H	OH	H	H

^a^: R = L-quinic acid.

**Table 5:**
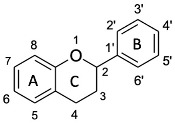
Different flavonoid chemical features contributing to the antioxidant activity of rice byproducts.

Compound	C3′	C4′	C5′	C2-C3	C3	C4	C5	C7
Quercetin	OH	OH	H	Double bond	OH	C=O	OH	OH
Myricetin	OH	OH	OH	Double bond	OH	C=O	OH	OH
Apigenin	H	OH	H	Double bond	H	C=O	OH	OH
Catechin	H	OH	OH	-	OH	H	OH	OH
Rutin	H	OH	OH	Double bond	O-R ^a^	C=O	OH	OH
Tricin	OCH_3_	OH	OCH_3_	Double bond	H	C=O	OH	OH
Luteolin	OH	OH	H	Double bond	H	C=O	OH	OH
Cyanidin-3-*O*-glucoside	OH	OH	H	Double bond	O-R ^b^	H	OH	OH
Cyanidin-3-*O*-rutinoside	OH	OH	H	Double bond	O-R ^a^	H	OH	OH
Cyanidin-3-*O*-galactoside	OH	OH	H	Double bond	O-R ^c^	H	OH	OH
Peonidin-3-*O*-glucoside	OCH_3_	OH	H	Double bond	O-R ^b^	H	OH	OH

^a^: R = rutinose; ^b^: R = glucose; ^c^: R = galactose.

**Table 6:**
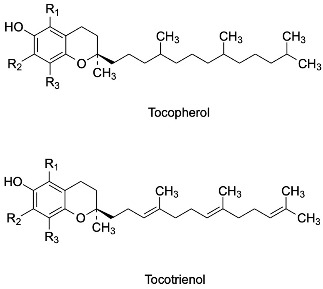
Structures of tocopherols and tocotrienols.

Vitamin E Isoform	R_1_	R_2_	R_3_
α-tocopherol	CH_3_	CH_3_	CH_3_
α-tocotrienol	CH_3_	CH_3_	CH_3_
β-tocopherol	CH_3_	H	CH_3_
γ-tocopherol	H	CH_3_	CH_3_
γ-tocotrienol	H	CH_3_	CH_3_
δ-tocopherol	H	H	CH_3_

## Data Availability

Data is contained within the article.
